# Analytical Applicability of Graphene-Modified Electrode in Sunset Yellow Electrochemical Assay

**DOI:** 10.3390/s23042160

**Published:** 2023-02-14

**Authors:** Lidia Măgeruşan, Florina Pogăcean, Bogdan Ionuţ Cozar, Stela Pruneanu

**Affiliations:** National Institute for Research and Development of Isotopic and Molecular Technologies, 67-103 Donat Street, 400293 Cluj-Napoca, Romania

**Keywords:** sunset yellow, graphene-modified electrode, electrochemical detection

## Abstract

Due to the recent increase in average living standards, food safety has caught public attention. It is necessary to conduct a qualitative and quantitative rapid test of prohibited food additives since the inclusion of food additives or the improper usage of synthetic dyes can negatively impact on the human health. Herein, a highly sensitive method for Sunset Yellow detection based on a glassy carbon electrode modified with few-layer graphenes was proposed. The electrochemical behavior of SY at the GR-exf/GCE modified surface was investigated by Cyclic Voltammetry, Square Wave Voltammetry, Electrochemical Impedance Spectroscopy and Amperometry. The influences of pH, scan rate, and interfering species were studied. Under optimized conditions, the developed sensor shows good linearity over a broad SY concentration range, e.g., 0.028–30 µM, with a low limit of detection (LOD = 0.0085 µM) and quantification (LOQ = 0.028 µM) (data obtained by amperometric technique). Furthermore, the modified electrode shows good selectivity, precision and sensitivity and has been successfully applied for SY quantification from commercially available pharmaceutical formulation as well as from candy bars and orange juice.

## 1. Introduction

The advent of food additives is an unavoidable result of the food processing industry expansion. Their usage in the food sector is essential, since it enables loss reduction, quality improvement, shelf life extension, the creation of novel formulas, and standardization, fulfilling the ever-changing demands of the market. Currently, there are more than 4000 different types of food additives used directly worldwide [1]. According to their raw material source and manufacturing processes, food additives can be classified as either synthetic or natural [2]. In order to boost specific quality and organoleptic features, in response to consumers’ growing demand, driven by the desire for items that are more appealing and aesthetically pleasing, food colorants have been extensively employed in the food processing industry, medicine, and cosmetics manufacture as one of the most significant food additives [3]. Nowadays, the majority of manufactured food colors are derived from crude oil or petroleum [4]. Thus, at a fraction of the expenses of obtaining and processing the resources needed to create natural colorings, synthetic dyes may be mass-produced; explaining their choice and extensive employment, beside their long-term stability compared to natural food coloring. However, their intense usage and consumption has been linked to a variety of adverse consequences, including: short-, medium- and long-term toxicity, allergic responses, respiratory affections, behavioral, and neurocognitive impacts on children (such as the development of attention deficit hyperactivity disorder—ADHD) as well as their possible carcinogenic effects [5,6]. 

Disodium 6-hydroxy-5-[(4-sulfophenyl)azo]-2-napthalenesulfononate) also known as Sunset Yellow FCF (SY) or E 110 is a petroleum-derived, water-soluble, orange azo dye, that is globally employed in a variety of food products (e.g., fruit juices, soft drinks, and other beverages, sauces, bakery, candies, chocolate, yoghurt, meat), cosmetics and pharmaceutical items. Along time, different studies revealed the potential harmful effect of SY dietary exposure on humans. Since its consumption in high amounts may trigger severe health complications and side effects (immune suppression, neurotoxicity, hepatocellular damage, renal failure, ADHD) [7,8], in many countries, SY usage is heavily restricted, and the acceptable daily intake dosage has been set by the European Food Safety Authority (EFSA) at 4 mg/kg [9].

In this general context, enhancing food safety and quality is essential for human welfare, and the intensive use of SY in the food sector is correlated with the need for developing trustworthy, accurate, sensitive, and selective analytical tools for its identification and quantification. Since azo dyes are generally distinguished by the presence of two types of active functional groups (azo bonds and hydroxyl groups), electrochemical tools may be easily employed for their quantitative detection. Compared to other analysis techniques and conventional analytical methods previously employed (e.g., UV-VIS spectroscopy [10]; electrochemiluminescence [11]; HPLC [12]), the electrochemical approach provides a rapid response, great sensitivity and good selectivity [13,14]. Furthermore, the employment of electrochemical detection demonstrated a potential use in the field of food safety analysis due to its portability, low power consumption and affordable equipment. 

Studies on the electrochemical detection of Sunset Yellow may be dated back to 2002; however, the majority of research on this area began in 2009. Different types of modified electrodes have been designed for this purpose: platinum wire-coated electrode [15]; boron-doped diamond [16]; polyallylamine [17]; poly(l-cysteine) [18]; multi-walled carbon nanotubes [19]; graphene and mesoporous TiO_2_ [20]; bimetallic nanoparticle-functionalized graphene [21]; graphene oxide [22], polyacrylamide membrane [23], carbon paper modified with graphite powder [24]; cuprous oxide-electrochemically reduced graphene oxide nanocomposites [25]; hierarchical flower-like NiCo_2_O_4_ nanoplates [26]; zinc oxide nanoflower [27]; multi-walled carbon nanotubes (MWCNTs) and electropolymerized 4-aminobenzoic acid (4-ABA) [28]; and poly(3,4-ethylenedioxythiophene):polystyrene sulfonate (PEDOT:PSS) [29]. Among all, the carbon-based materials, especially graphene, hold great potential in electrode modification due to the extraordinary and versatile intrinsic and extrinsic properties [30,31]. Over time, a variety of methods, either bottom–up or top–down, have been applied for graphene production [32,33], but the obtaining of good quality few-layer materials in an efficacious and environmentally friendly manner is still of great interest. Thus, the development of effective processes for graphene production by graphite electrochemical exfoliation without the use of organic solvents is of particular relevance in this context. 

The major goal of this work is to provide an electrochemical approach for the quick, easy, and sensitive detection of Sunset Yellow with a glassy carbon electrode modified with few-layer graphenes produced by graphite rod electrochemical exfoliation via pulses of current. The novelty of the developed sensor is related to its ability to detect SY in a variety of real samples (pharmaceutical drug; candy bar; orange juices) containing many interfering species (e.g., food additives and other azo dyes), proving its practical applicability.

## 2. Materials and Methods

### 2.1. Chemicals

Every chemical utilized was of the analytical grade and was not further purified before usage. Graphite rods (6 mm diameter, 99.995% purity), potassium chloride (KCl, 99.98%), Sunset Yellow FCF (SY, dye content 90%) and Tartrazine (TRZ, dye content ≥ 85%) were purchased from Sigma-Aldrich (Kandel, Germany). Sodium dihydrogen phosphate (NaH_2_PO_4_, 100%) and di-sodium hydrogen phosphate anhydrous (Na_2_HPO_4_, 99.7%) were supplied from VWR Chemicals (Leuven, Belgium). Sodium acetate anhydrous (CH_3_COONa, ≥99.0%) and ammonium sulfate (NH_4_)_2_SO_4_, ≥99.0%) were provided from REACTIVUL Bucuresti (Romania). Potassium ferrocyanide K_4_[Fe(CN)_6_, L(+)-Ascorbic acid (C_6_H_8_O_6_, AA, ≥99%), and citric acid (C₆H₈O₇, CA, ≥99.5%) were acquired from Merck (Darmstadt, Germany). N,N-dimethylformamide (DMF) and ammonium thiocyanate (NH_4_SCN, ≥99%) were acquired from Fluka Chemie GmbH (Buchs, Switzerland). Deionized water with a resistivity of at least 18.2 MΩ × cm was used to prepare all solutions. 

### 2.2. Apparatus

In order to reveal the morphological characteristics of the graphene sample, we employed a Hitachi HD2700 instrument (Hitachi, Tokyo, Japan) equipped with a cold field emission gun (CSEG). The structural characterization of graphene was performed by X-ray powder diffraction (XRD). A Bruker D8 Advance Diffractometer (40 kV, 0.5 mA) equipped with an LYNXEXE detector (λ = 1.5406 Å) was employed. Raman spectra were recorded with an NTEGRA Spectra platform, which was placed on a NEWPORT RS4000 optical table and equipped with a SOLAR TII confocal Raman spectrometer coupled with an Olympus IX71 microscope in two different configurations (Moscow, Russia). The sample obtained after the electrochemical exfoliation of graphite rods was freeze-dried with Christ-Alpha 1-4 LSC equipment (Germany). All the electrochemical measurements (Cyclic Voltammetry—CV; Square Wave Voltammetry—SW, Amperometry—AMP and Electrochemical Impedance Spectroscopy—EIS) were recorded with a Potentiostat/Galvanostat Instrument (PGSTAT-302N, Metrohm-Autolab B.V., Utrecht, The Netherlands). 

### 2.3. Synthesis of Graphene Sample by Exfoliation of Graphite Rods (GR-exf) 

Two graphite rods were employed as anode and cathode in an electrochemical cell filled with 0.1 M ammonium sulfate and 0.1 M ammonium thiocyanate and connected to the exfoliation system. The exfoliation system generated current pulses and the time parameters of the pulses, such as the pulse duration (0.8 s) and the pause between two pulses (0.2 s), were set before starting the graphene synthesis [34]. A bias of 9 V was applied between the anode and cathode, and after 3 h, the exfoliation process was stopped. In order to remove the ions present in solution and some of them superficially attached to graphene, the resulting material was washed with 10 L of distilled water, filtered with Whatman qualitative paper (white-ribbon filter) and dried by lyophilization. The sample was following denoted GR-exf. 

### 2.4. Modification of Electrode Surface with Graphene (GR-exf/GC)

An organic solvent (N,N-dimethylformamide—DMF) was chosen for the dispersion of graphene sample (2 mg/mL) due to its high boiling point (153 °C). As a consequence, it evaporates slower at room temperature, allowing the graphene flakes to interact by π–π stacking with the glassy–carbon (GC) surface, forming a stable layer on top of the electrode. A volume of 10 µL from the graphene dispersion in DMF was deposited by drop-casting on top of GC and dried at room temperature for 24 h. As expected, the addition of various amounts of graphene on top of the GC electrode leads to the increase in both faradaic and capacitive currents; therefore, an optimum amount has to be found. In our case, for the oxidation of SY, this amount was 10 µL (see Figure S1 in Supplementary Materials). Both the bare glassy carbon and the modified electrode (GR-exf/GC) were used for SY electrochemical detection and quantification, and their performances were compared.

### 2.5. Real Sample Analysis with Graphene-Modified Electrode

In order to prove the practical applicability of the graphene-modified electrode in real sample analysis, we chose a variety of samples such as pharmaceutical drug (triferment) candy bar and orange juices.

Triferment tablets are used in non-digestive disorders due to the insufficient secretion of pancreatic enzymes (e.g., exocrine pancreatic insufficiency). Triferment contains the active substance pancreatin made up of pancreatic enzymes (lipase, amylase, and protease). One gastro-resistant tablet contains 275 mg of pancreatin. The other components in the tablet core are: corn starch, talc and sugar. The surrounding film (orange color) is composed by: hypromellose 15 cP (E 464), lactose monohydrate, titanium dioxide (E 171), macrogol 4000, citric acid monohydrate (E 330), methacrylic acid copolymer type C, talc, triethyl citrate (E 1505), colloidal silicon dioxide anhydrous, sodium hydrogen carbonate (E 500), sodium lauryl sulfate, partially hydrolyzed polyvinyl alcohol, macrogol 3350, Ponceau 4R (E 124), and Sunset Yellow FCF (E 110).

A lollipop candy bar containing sugar, glucose, citric acid (E330) as well as tartrazine (E102), Sunset-Yellow (E110), azorubine (E122) and brilliant blue (E133) was employed as a food sample. Furthermore, two commercially available orange juices were analyzed. The first orange juice contained the following: orange juice from concentrate (3%); carbon dioxide; citric acid; malic acid; sucralose; neohesperidin DC (E959); glucosides; potassium sorbate; guar gum; ascorbic acid; orange flavors; and carotene dye. As labeled, the ingredients for the second orange juice sample were the following: water; sugar; orange juice from concentrate (4%); carbon dioxide; citric acid; orange flavors; arabic gum; sucrose acetate isobutyrate (E444); ascorbic acid; potassium sorbate; carotene dye; glycerol esters.

## 3. Results and Discussion

### 3.1. Morphological and Structural Investigation of Graphene Sample

**Figure 1:**
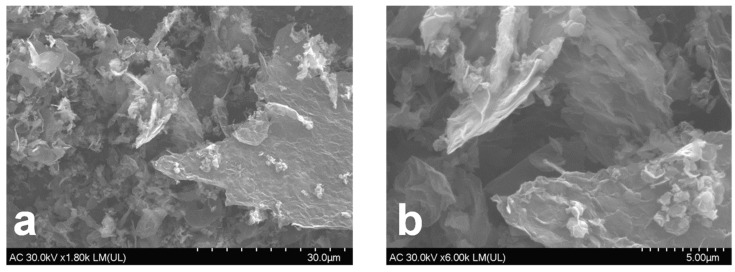
SEM micrographs of graphene sample, showing the porous morphology of the deposited layer: scale bar 30 µm (**a**) and 5 µm (**b**).

Representative SEM micrographs of graphene sample obtained after the exfoliation process are shown in Figure 1. Some of the flakes are very large (e.g., 10–30 µm length) and have a wavy surface, while others are smaller (2–3 µm). It is important to mention that the deposited graphene layer has a porous morphology, and therefore, we can expect a larger active area for the modified electrode in comparison with bare glassy carbon.

**Figure 2:**
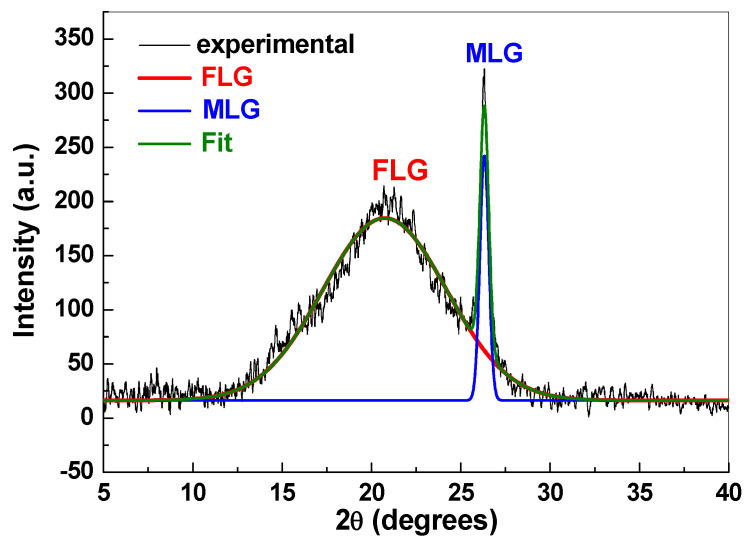
The XRD pattern of graphene showing the FLG and MLG peaks.

The XRD pattern of graphene (Figure 2) reveals more important information: that the material is a mixture of few-layer graphene (FLG) and multi-layer graphene (MLG). The two peaks associated to FLG and MLG reflections can be seen at 20.78° and 26.31°, respectively. Similar with graphite, MLG are formed due to the strong π–π stacking interaction between single layers that helped them to increase their thermodynamic stability [35]. However, the majority of MLG was removed during the washing and filtration procedure, so FLG is predominant within the sample (91.27% in comparison to 8.73% for MLG). Other structural parameters associated with graphene were determined, such as the average number of layers present within the graphene crystallites (n); the mean size of graphene crystallite (D) and the interlayer spacing (d) [36] (see the summarization of data presented in Table 1).

Raman spectroscopy was employed to study the structural disorder degree in the exfoliated graphene sample. As can be seen in Figure S2, all the characteristic Raman bands of graphene are present: the most intense one is the defect band (D) at ~1353 cm^−1^ which appears due to the structural defects in the sp^2^ hybridized carbon network; the graphite band (G) appears at ~1605 cm^−1^ and is a primary in-plane vibration mode of the sp^2^ hybridized carbon network, the 2D band appears at ~2700 cm^−1^ being a second-order overtone of different in-plane vibrations, and the D+G band (2940 cm^−1^) is a combination of scattering peaks. It is important to mention that the intensity of the D band is higher than that of the G band, indicating that defects are present in the graphene lattice. 

According to Cançado et al. [37], the I_D_/I_G_ ratio is related to the in-plane crystallite size (L_a_) of graphene (Equation (1)) and gives an indication of the defect-free domains:

(1) $$L_{a}\left(\mathrm{nm}\right)=\frac{560}{E_{l}^{4}}{\left(\frac{I_{D}}{I_{G}}\right)}^{-1}$$

where E_l_ represents the laser excitation energy (2.33 eV). 

In our case, L_a_ was determined to be 15.56 nm.

### 3.2. Electrochemical Studies

In order to study the electrochemical behavior of the Gr-exf/GC electrode toward SY detection, it was necessary to determine the active surface area of the modified electrode. Thus, cyclic voltammetry experiments were performed at different scanning rates, in the range of 2–100 mV/s, in the presence of 10^−3^ M redox indicator potassium hexacyanoferrate(II)—K_4_Fe(CN)_6_ (0.2 M KCl supporting electrolyte), and the electrode area was calculated using the Randles–Ševcik equation [38] (see Figure S3a,b from Electronic Supplementary Materials). As can be seen in Figure S3b, the peak intensity versus the square root of the scan rate follows the linear regression equation defined as: Ip = 2.89 × 10^−7^ + 2.98 × 10^−5^ × ʋ^1/2^ (R^2^ = 0.997). When compared to the original bare GC electrode, the graphene-modified electrode’s surface area is noticeably larger: 0.044 cm^2^ compared to 0.028 cm^2^.

In addition, electrochemical impedance spectroscopy was employed to determine the charge-transfer resistance of each electrode. The EIS spectra for GC and GR-exf/GC can be seen in Figure S4, and they were interpreted based on Randles equivalent electrical circuit (inset) [39]. In the case of a GC electrode (Figure S4-blue), the circuit contains the solution resistance (R_s_), the Warburg impedance (Z_W_), which characterizes the diffusion of ions toward the interface, the charge-transfer resistance (R_ct_) which indicates the easiness of electron transfer across the interface and the double layer capacitance (C_dl_). For the GR-exf/GC electrode (Figure S4-red), the C_dl_ and Z_W_ components were replaced by Constant Phase Elements (CPE) due to the roughness of the surface. According to Brett et al., CPE arises due to the roughness of the electrode surface and dynamic disorder associated with the diffusion [40]. After fitting the experimental data with the proposed equivalent electrical circuits, the R_ct_ values for a bare and graphene-modified electrode were determined. For a bare GC, we obtained a very large R_ct_ value (80 kΩ), indicating a slow transfer of electrons at the electrode/solution interface. On the other hand, GR-exf/GC had a considerably lower value for R_ct_ (28 Ω), indicating an easy electron transfer during the redox process.

**Figure 3:**
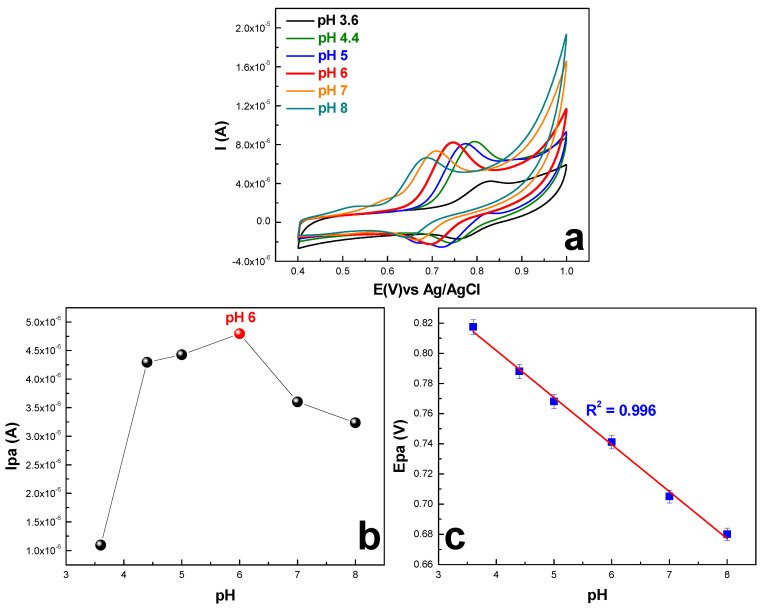
(**a**) CVs recorded with GR-exf/GC in buffer solutions containing 100 µM SY at different pH values (3.6; 4.4; 5.0; 6.0; 7.0 and 8.0); scan rate: 10 mV/s; (**b**) Anodic peak current versus pH value; (**c**) Plot of anodic peak potential versus pH value.

The pH effect on the electrochemical response of SY on a GR-exf/GC-modified surface was studied by CV measurements in acetate and phosphate buffer solutions containing 100 µM SY in the 3.6–8 pH range. As depicted in Figure 3a, the pH solution value had a significant influence on both the peak potential and peak current of SY. The oxidation peak current increased with the increase in pH value until reaching its maximum at pH 6, and then, it decreased gradually as the pH continued increasing (see Figure 3b). Therefore, to achieve the sensitive determination for SY, the pH 6 PBS buffer solution was chosen as the optimal electrolyte for the subsequent analytical experiments. Furthermore, oxidation peak potential shifts linearly to more negative potential as the pH value increases, demonstrating that protons are involved in the electrode reaction of SY at the surface of the GR-exf/GC-modified electrode [18]. 

The linear relationship between anodic peak potential and pH value (Figure 3c) follows the linear regression equation: E_pa_ = 0.909–0.028∙pH (R^2^ = 0.996). The slope (28 mV/pH) is almost half of the theoretical Nernstian value of 59 mV/pH [41]. From the obtained data, it may be inferred that Sunset Yellow undergoes a process of oxidation with the transfer of protons and electrons in a ratio of 1:2. 

**Figure 4:**
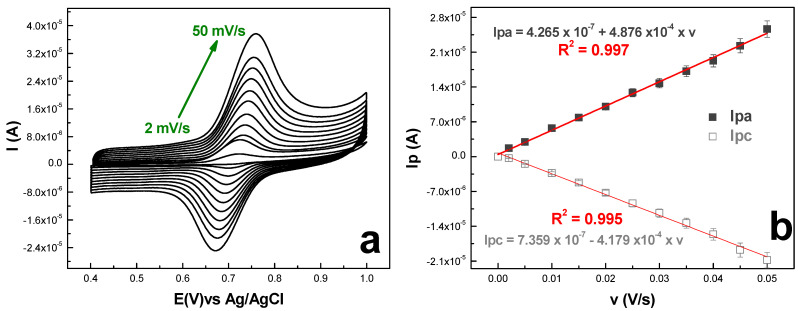
(**a**) CV measurements obtained at GR-exf/GC-modified electrode surface in 100 µM SY at (PBS, pH 6) under various scan rates (2–50 mV/s); (**b**) Intensity of anodic and cathodic peaks versus scan rate.

To further investigate the SY reaction at the surface of the GR-exf/GC-modified electrode, CV measurements were performed under various scan rates (ranging between 2 and 50 mV/s) in PBS buffer solution (pH 6) containing 100 µM SY. As depicted in Figure 4a, with the gradual increase in the scan rates, the oxidation peak shifted to more positive potential, while the reduction peak shifted to more negative potential. Furthermore, the initial ΔEp value (≈0.015 V) increases with the scan rate up to ≈0.083 V (recorded when the the scan rate was 50 mV/s). Thus, the system is neither irreversible nor reversible, taking into account the current differences, but it can be considered rather quasi-reversible [42]. The scan rate dependence of anodic and cathodic peak currents follows the linear regression equations described as: I_pa_ = 4.265 × 10^−7^ + 4.876 × 10^−4^ × v and I_pc_ = 7.369 × 10^−7^ − 4.179 × 10^−4^ × v, respectively (Figure 4b). These findings suggest that the electro-oxidation of the SY molecule is an adsorption-controlled process [43], which is in good agreement with the previous reported studies of Nguyen et al. [44] and Wang et al. [45]. 

**Scheme 1:**
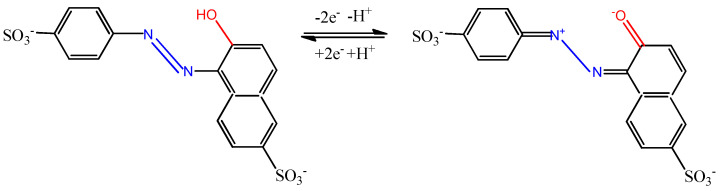
Sunset Yellow Electrochemical Oxidation Mechanism.

To date, we have been unable to find a totally accepted theory regarding the electro-oxidation pathway of the SY molecule. Many of the previously reported mechanisms are described by irreversible electrode reactions which imply the involvement of an equivalent amount of protons and electrons [46,47,48,49], the hydroxyl group being considered responsible for the electrode reaction, undergoing a transformation to carbonyl. However, there are several reports which take into account an unequal number of protons and electrons for the SY electro-oxidation mechanism, among which it is worth mentioning the works of Chebotarev et al. [43] and Nguyen et al. [44]. Based on the obtained results, a possible SY electro-oxidation pathway at the GR-exf/GC-modified electrode surface was proposed and is depicted in Scheme 1. 

**Figure 5:**
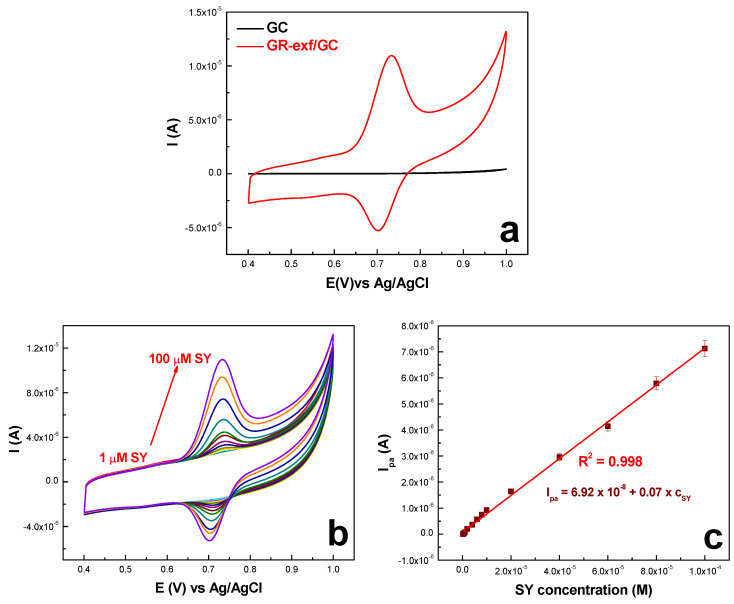
(**a**) Cyclic voltammograms recorded with: bare GC (black line) and GR-exf/GC (red line) in pH 6 PBS solution containing 100 µM SY; (**b**) CVs recorded with GR-exf /GC-modified electrode in PBS (pH 6) containing different SY concentration (1–100 µM), scan rate 10 mV/s; (**c**) corresponding calibration curve (peak intensity vs. SY concentration).

In order to assess the electrochemical response of both unmodified and graphene-modified surfaces, cyclic voltammetry measurements were recorded in pH 6 PBS solution containing 100 µM SY (see Figure 5a). In the case of bare GC, no electrochemical response was observed, while the SY electro-oxidation at the graphene-modified surface generates a well-defined oxidation response at +0.76 V, which is accompanied by a small reduction peak at +0.64 V. As depicted in Figure 5b, the oxidation peak current demonstrates a rising trend over a SY concentration range of 1–100 μM, with a linear dependence of the anodic peak current on the analyte concentration (Figure 5c) described by: I_pa_ = 6.92 × 10^−8^ + 0.07 × C_SY_ (R^2^ = 0.998). The limit of detection (LOD) and the limit of quantification (LOQ) were determined to be 0.303 µM and 1 µM, respectively.

**Figure 6:**
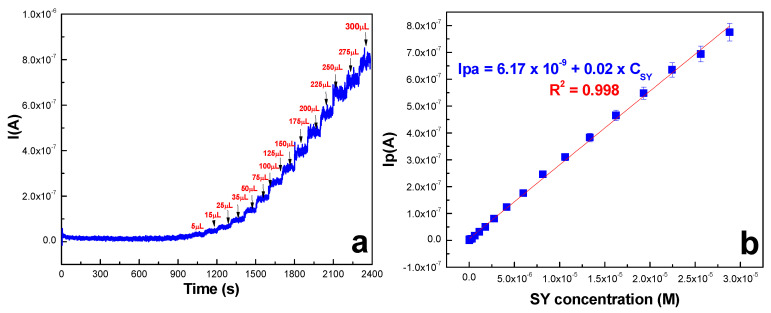
(**a**) Amperometric curves recorded with GR-exf/GC (+0.75 V applied potential) in pH 6 supporting electrolyte, after the addition of SY from 10^−4^ M stock solution; the SY concentration range was 0.028–30 µM; (**b**) the corresponding calibration plot obtained with background subtracted signals.

The use of an amperometric method (+0.75 V applied potential) led to better results, as can be seen in Figure 6. In this case, the SY linear concentration range was from 0.028 to 30 µM (regression equation I_pa_ = 6.17 × 10^−9^ + 0.02 × C_SY_; R^2^ = 0.998), and the detection limit (0.0085 µM) was lower by comparison with that obtained from CV measurements (0.303 µM). 

In the case of using square wave voltammetry (Figure S5), the obtained results were similar to those resulting from CV measurements: the SY linear concentration range was from 1 to 100 µM, with the linear regression equation I_pa_ = −1.43 × 10^−7^ + 0.15 × C_SY_ (R^2^ = 0.993) and the detection limit (0.303 µM) was similar to that obtained from CV measurements. However, the sensitivity (0.15 A/M) was two times higher in comparison with that obtained by CV (0.07 A/M).

The developed sensor’s analytical features were compared to those of the previously reported electrodes (see Table 2). As can be seen, the findings of this study indicate that the GR-exf/GC sensor exhibits equivalent and in some cases even better characteristics than those of other studies, demonstrating the added value of the electrode coated with graphene-based material. The improved electrocatalytic activity may be linked to the increase in the electron transfer rate at the electrolyte/electrode interface attributed to the structure and characteristics of the few-layer graphene material, which facilitates the SY oxidation.

The modified surface stability during measurements was tested by subjecting the working electrode to 50 successive scans in PBS solution (pH 6) containing 100 µM SY (see Figure S6a from Electronic Supplementary Materials). It was observed that at the end of the last measured voltammetric cycle, the modified electrode retains 83.56% of its original signal, suggesting a fair stability of the GR-exf/GC sensor during measurements. Furthermore, the intra-day and inter-day reproducibility of the method was tested by carrying out experiments, in similar conditions, on the same modified electrode. The differences between measurements performed in the same day do not exceed 5.87%. The modified electrode-sensing capacities were tested from time to time over a large time period (more than 3 months), and it was observed that the electrode retains 83.07% of its initial signal after 120 days of storage at room temperature (see Figure S6b from the Electronic Supplementary Materials). 

**Figure 7:**
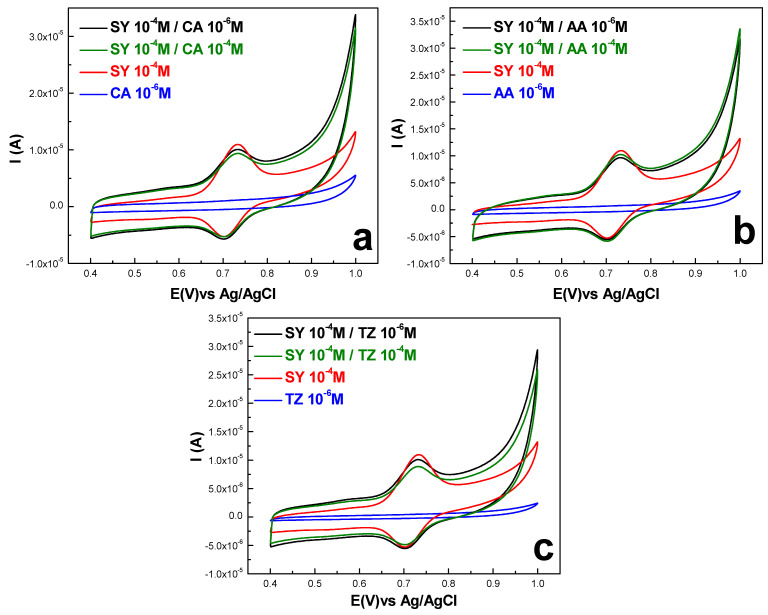
(**a**) CV response of GR-exf/GC-modified electrode toward 100 µM SY in the presence of different organic interferences set at two fixed concentrations of 1 µM and 100 µM: (**a**) citric acid (CA); (**b**) ascorbic acid (AA); (**c**) tartrazine (TZ). Supporting electrolyte pH 6 PBS, scanning rate 10 mV/s.

In addition, the SY detection and quantification at GR-exf/GC surface was performed in the presence of some interfering species that can be found together with SY in a food and pharmaceutical matrix. For exemplification, in Figure 7, the results obtained in the presence of citric acid (CA), ascorbic acid (AA) and tartrazine (TZ) are presented. First, the interference level was set at 1 µM, while the concentration for the analyte of interest was 100 times higher (100 µM). As visible from the blue curves in Figure 7a–c, no signal was generated by the organic interfering species at the developed sensor surface. Afterwards, equal amounts of interferences and SY were present in the analyzed solution (100 µM), and the peak signal was affected by such high concentration mainly in the case of tartrazine. According to the World Health Organization (WHO) safety regulations, the maximum amount of accepted azo dyes in food and drinks should not exceed 10^−4^ M. Our investigation of SY content in pharmaceutical drug, food and drinks proved a good selectivity of the developed sensor, and the SY signal was detected in the complex matrices of the analyzed samples (see next paragraph).

**Figure 8:**
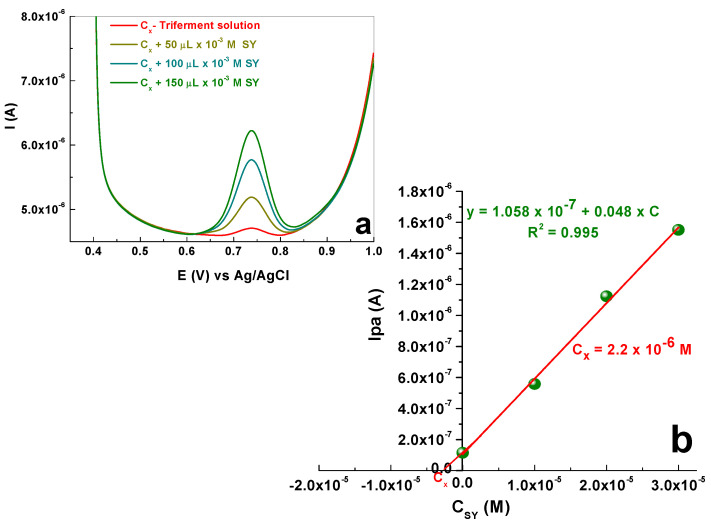
Square-wave voltammograms recorded with GR-exf/GC-modified electrode in Triferment solutions (**a**); the standard addition plot which allowed the determination of C_x_ in Triferment tablet (**b**).

In order to test the validity of the SY detection method, the analysis was performed in real sample solution. Triferment was selected from the pharmaceutical drugs that contain the artificial colorant, Sunset Yellow. Since in this case, SY was present only in the film surrounding the tablet, the following procedure was applied. One tablet was immersed in a beaker containing 5 mL of pH 6 PBS and left in the solution until the film was completely dissolved (the tablet core was removed from the solution after film dissolution). This was considered the stock solution for SY in Triferment. A volume of 200 µL from the stock solution was mixed with 4.8 mL pH 6 PBS and denoted the unknown concentration (C_x_) of SY in Triferment. Next, three volumes (e.g., 50, 100 and 150 µL) of SY standard solution (10^−3^ M in pH 6 PBS) were added to three beakers, each also containing C_x_ (the final volume in the beakers was 5 mL). The electrochemical signal corresponding to each solution was recorded with a GR-exf/GC-modified electrode (Figure 8a), and then, the peak current (I_pa_) was plotted versus SY concentration. From the obtained calibration plot (Figure 8b), the C_x_ concentration was determined to be 2.2 µM. By taking into account that C_x_ was 25 times diluted from the stock solution, the SY concentration in the stock was determined to be 55 µM.

Furthermore, a lollipop candy bar was employed as the food sample. The concentration of SY in the lollipop candy was determined as described in the following. A quantity of 186 mg lollipop candy (sugar + SY colorant) was dissolved in 2 mL pH 6 PBS, and the SY concentration was denoted as C_x_. After the complete dissolution of the candy, known volumes from SY stock solution (10^−3^ M) were added in the same beaker (e.g., 10, 20, 40 and 70 µL), and the corresponding oxidation signals were recorded with a GR-exf/GC-modified electrode (Figure S7a). From the obtained calibration plot (peak signal versus SY added concentration), the C_x_ concentration of SY was determined to be 7.44 µM (Figure S7b). 

In addition, the SY concentration in the first commercial orange juice solution was determined according to the following procedure: a volume of 10 mL orange juice was mixed with 10 mL distilled water, and the solution pH was brought to pH 6 by adding the corresponding phosphate salts. Then, 2 mL from the diluted orange juice was put in a beaker, and next, known volumes from SY stock solution (10^−3^ M) were added in the same beaker (e.g., 20, 40 and 70 µL) and the corresponding oxidation signals were recorded with a GR-exf/GC-modified electrode (Figure S8a). From the obtained calibration plot (peak signal versus SY added concentration), the C_x_ concentration of SY in orange juice was determined to be 2.77 µM (Figure S7b). Since the orange juice was two times diluted with water, the real concentration from the juice was C_real_ = 2C_x_ = 5.54 µM.

The SY concentration in the second commercial orange juice solution was also determined. First, a volume of 10 mL orange juice was mixed with 30 mL of distilled water, and the solution pH was brought to pH 6 by adding the corresponding phosphate salts. Then, 2 mL from the diluted orange juice was put in a beaker, and next, known volumes from SY stock solution (10^−3^ M) were added in the same beaker (e.g., 20, 40 and 70 µL), and the corresponding oxidation signals were recorded with a GR-exf/GC-modified electrode (Figure S9a). From the obtained calibration plot (peak signal versus SY added concentration), the C_x_ concentration of SY in the second orange juice was determined to be 2 µM (Figure S9b). Since the orange juice was four times diluted, the real concentration from the juice was C_real_ = 4C_x_ = 8 µM.

## 4. Conclusions

In this paper, we presented the results obtained with a glassy carbon electrode having its surface modified with few-layer graphenes produced by the electrochemical exfoliation of graphite rods. Its applicability in Sunset Yellow detection and quantification was tested. The electrochemical results showed that the modified electrode surface exhibits remarkable enhancement of the catalytic activity toward the SY electro-oxidation compared to the bare electrode surface. In pH 6 PBS, a quasi-reversible process occurs over a wide SY concentration range (1–100 µM), and the anodic peak intensity is linearly proportional with SY concentration. The limit of detection was determined using cyclic voltammetry, square wave voltammetry and amperometric approaches and was found to be 0.303 µM (from CV and SW) and 0.0085 µM (from amperometry). The developed sensor has been thoroughly validated and shows good anti-interferences capacity, stability, precision and sensitivity. The GR-exf/GC sensor applicability in real sample analysis was tested with good accuracy, demonstrating its usefulness in the SY detection. 

## Figures and Tables

**Table 1:** The XRD parameters associated to Gr-exf material.

Graphene	2Θ (Degrees)	D (nm)	n	d (nm)	Amount (%)
FLG	20.78	1.23	3	0.43	91.27
MLG	26.31	19.27	56	0.334	8.73

**Table 2:** Comparison with other reported electrochemical sensors for SY detection.

Electrode	Method	Linear Range (µM)	Limit of Detection (µM)	Reference
**Metal oxides/GCE** GCE—glassy carbon electrode	DPV	19–270	5.7	[50]
**MWNT/GCE** MWNT—multi-walled carbon nanotubes GCE—glassy carbon electrode	DPV	0.055–11	0.022	[19]
**PLPA/GCE** PLPA—poly(L-phenylalanine) GCE—glassy carbon electrode	DPV	40–140	4	[51]
**MWCNT-GCE** MWNT—multi-walled carbon nanotubes GCE—glassy carbon electrode	DPV	0.55–7	0. 12	[52]
**ZnO-cysteic acid/GCE** GCE—glassy carbon electrode	DPV	0.1–3.0	0.03	[53]
**nAu/CPE** nAu—gold nanoparticles CPE—carbon-paste electrode	DPV	0.1–2	0.03	[54]
**MWCNTs–IL/CCE** MWCNTs–IL—multi-walled carbon nanotubes–ionic liquid CCE—Carbon–ceramic electrode	CV	0.4–110	0.1	[55]
**rGO/CPE** rGO—reduced graphene oxide CPE—carbon-paste electrode	CV	0.05–10	0.27 nM	[56]
**CTAB/MMT-Ca/CPE** CTAB/MMT-Ca—montmorillonite calcium (MMT-Ca) functionalized with cetyltrimethylammonium bromide (CTAB) CPE—carbon–paste electrode	CV	2.5 to 200 nM	0.71 nM	[57]
**GR-exf/GC**	CV	1–100	0.303	current study
**GR-exf/GC**	SW	1–100	0.303	current study
**GR-exf/GC**	Amp	0.028–30	0.0085	current study

## Data Availability

Data will be provided upon reasonable request to the corresponding author.

## Supplementary Materials

The following supporting information can be downloaded at: https://www.mdpi.com/1424-8220/23/4/2160/s1, Figure S1. Variation of SY peak current with the amount of graphene deposited on top of GC electrode (graphene concentration in DMF: 2 mg/mL); Figure S2. The Raman spectrum of exfoliated graphene-sample; Figure S3. (a) Cyclic voltammetric response of GR-exf/GC surface in the presence of 10^-3^ M redox indicator K_4_[Fe(CN)_6_] at various scanning rates (0.2 M KCl supporting electrolyte); (scan rates from 2 to 100 mV/s); (b) Linear plot of anodic peak current (I_p_) *vs* ʋ^1/2^; Figure S4. The EIS spectrum of GC (blue) and GR-exf/GC (red) electrodes recorded in 10^-3^ M K_4_[Fe(CN)_6_] solution (0.2 M KCl supporting electrolyte); *Inset:* the equivalent electrical circuits used to fit the impedance spectrum of GC and GR-exf/GC; Figure S5. (a) SWV recorded with GR-exf /GC electrode in PBS (pH 6) containing different SY concentration (1–100 µM); scan rate 10 mV/s; (b) the corresponding calibration curve (peak current vs. SY concentration); Figure S6. (a) 50 CVs recorded with GR-exf/GC modified electrode at a scan rate of 10 mV/s in PBS pH 6 solution containing 100 µM SY; (b) Anodic peak current intensity obtained in replicate CV measurements at GR-exf/GC surface over a long time interval of 120 days (100 µM SY solution, PBS pH 6); Figure S7. (a) Square wave voltammograms recorded with GR-exf/GC modified electrode in the lollipop solutions; (b) the standard addition plot which allowed the determination of C_x_ in lollipop solution; Figure S8. (a) Square wave voltammograms recorded with GR-exf/GC modified electrode in solutions containing orange juice 1; (b) the standard addition plot which allowed the determination of C_x_ in the first orange juice solution; Figure S9. (a) Square wave voltammograms recorded with GR-exf/GC modified electrode in solutions containing orange juice 2; (b) the standard addition plot which allowed the determination of C_x_ in the second orange juice solution.
