# Supplementary material for: Analytical Applicability of Graphene-Modified Electrode in Sunset Yellow Electrochemical Assay
Source: Sensors (Basel). 2023 Feb 14;23(4):2160. doi: 10.3390/s23042160 (PMC10004213; doi:10.3390/s23042160)
Supplement: Supplementary file 1 [file sensors-23-02160-s001.zip › sensors-2185896-supplementary.pdf]

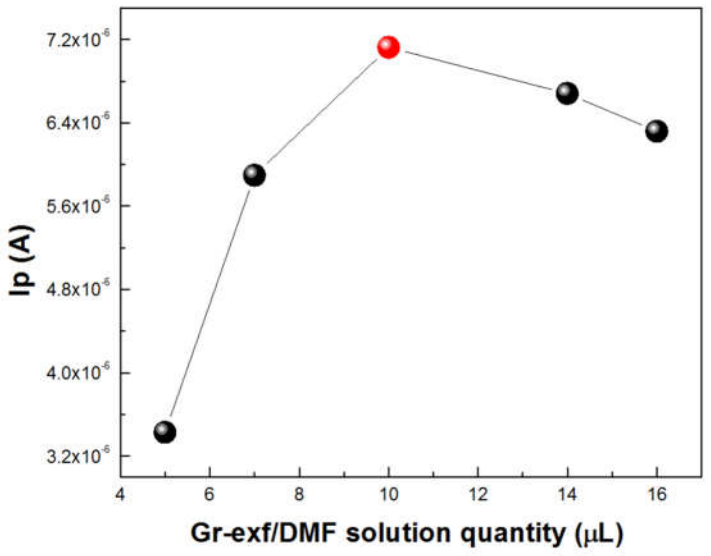

Figure S1. Variation of SY peak current with the amount of graphene deposited on top of GC electrode (graphene concentration in DMF: 2 mg/mL).

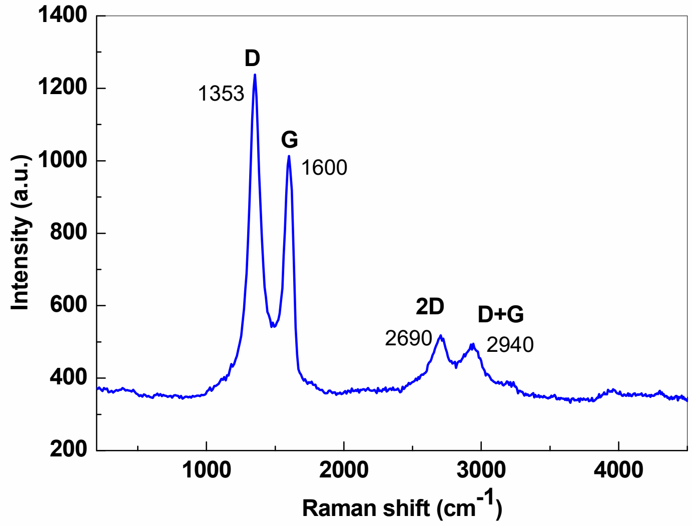

Figure S2. The Raman spectrum of exfoliated graphene-sample.

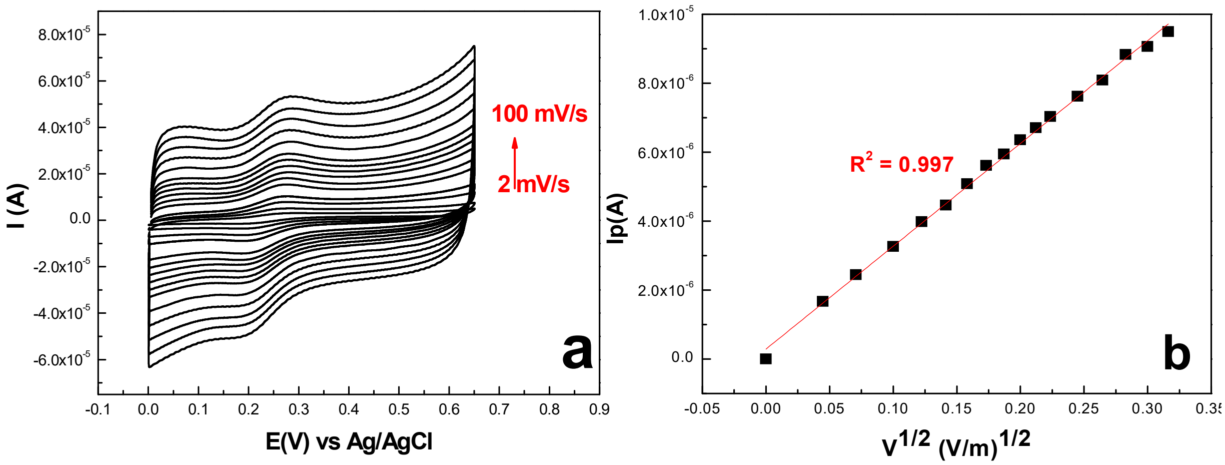

Figure S3. (**a**) Cyclic voltammetric response of GR-exf/GC surface in the presence of 10^{-3} M redox indicator $K_4[Fe(CN)_6]$ at various scanning rates (0.2 M KCl supporting electrolyte); scan rates from 2 to 100 mV/s); (**b**) Linear plot of anodic peak current (I_p) *vs* $\upsilon^{1/2.}$

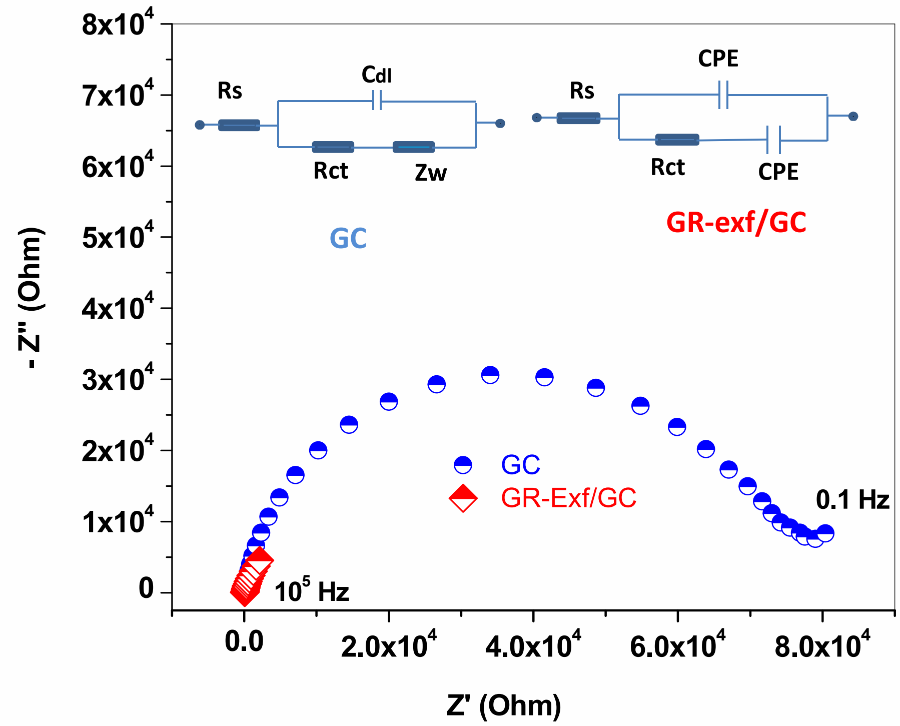

Figure S4. The EIS spectrum of GC (blue) and GR-exf/GC (red) electrodes recorded in 10^{-3} M $K_4[Fe(CN)_6]$ solution (0.2 M KCl supporting electrolyte); *Inset:* the equivalent electrical circuits used to fit the impedance spectrum of GC and GR-exf/GC.

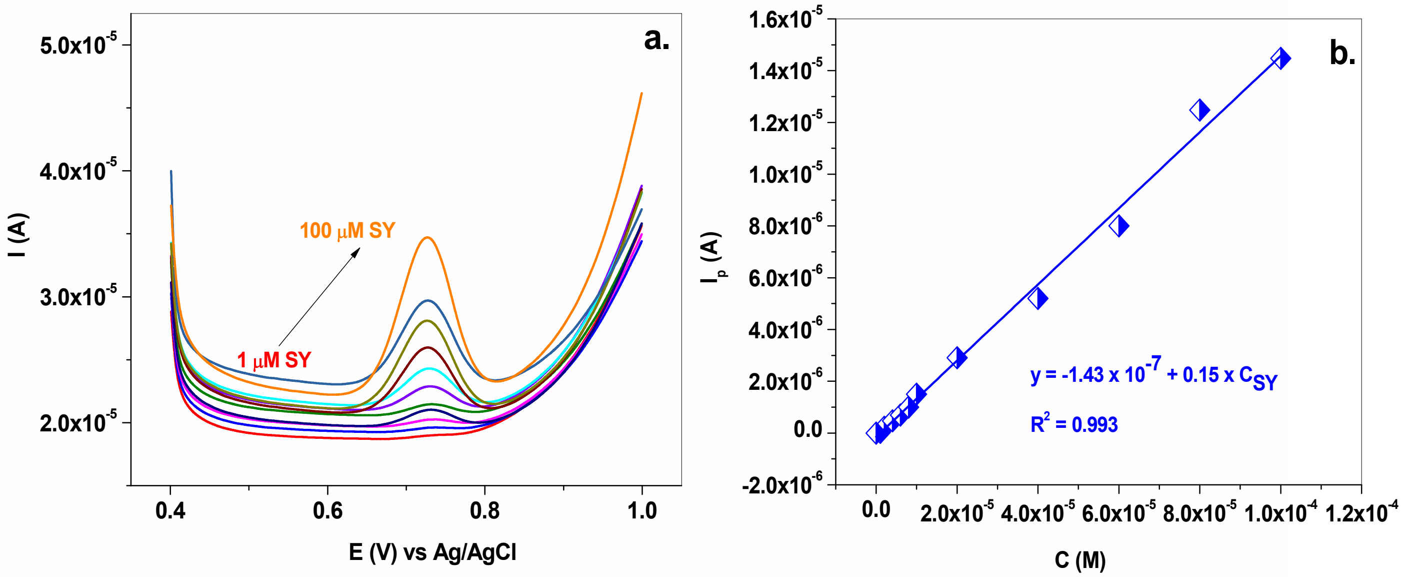

Figure S5. (**a**) SWV recorded with GR-exf /GC electrode in PBS (pH 6) containing different SY concentration (1–100 μM); scan rate 10 mV/s; (**b**) the corresponding calibration curve (peak current vs. SY concentration).

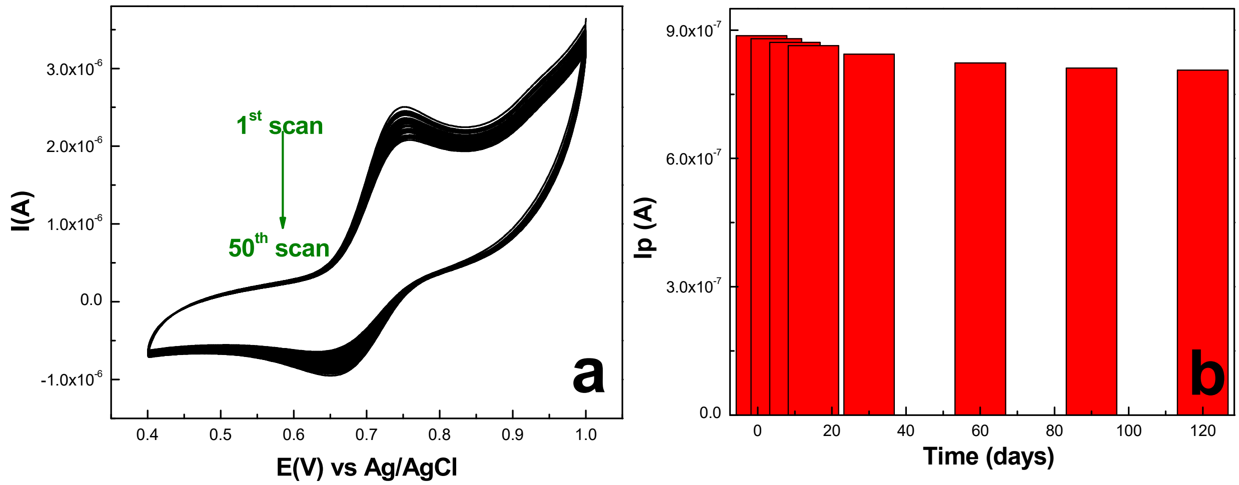

Figure S6. (**a**) 50 CVs recorded with GR-exf/GC modified electrode at a scan rate of 10mV/s in PBS pH 6 solution containing 100μM SY; (**b**) Anodic peak current intensity obtained in replicate CV measurements at GR-exf/GC surface over a long time interval of 120 days (100 μM SY solution, PBS pH 6).

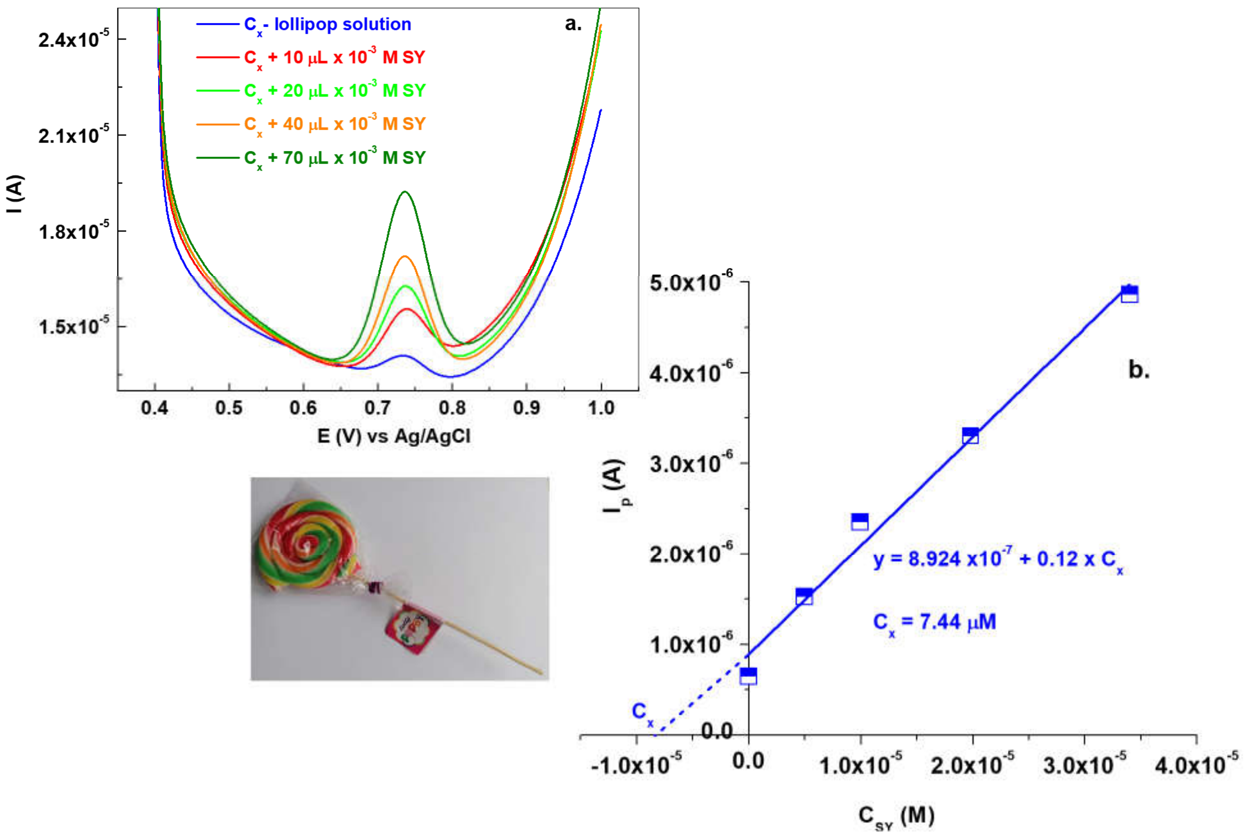

Figure S7. (**a**) Square wave voltammograms recorded with GR-exf/GC modified electrode in the lollipop solutions; (**b**) the standard addition plot which allowed the determination of C_x in lollipop solution.

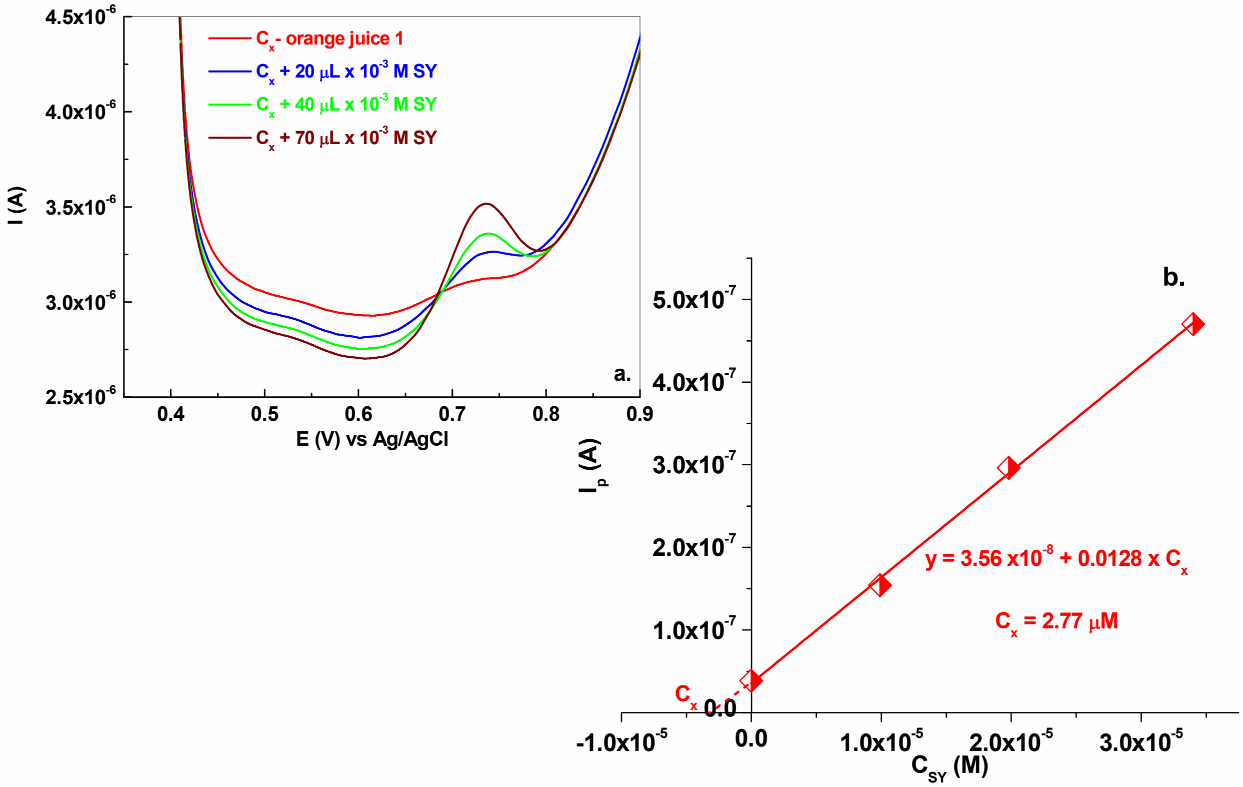

Figure S8. (**a**) Square wave voltammograms recorded with GR-exf/GC modified electrode in solutions containing orange juice 1; (**b**) the standard addition plot which allowed the determination of C_x in the first orange juice solution.

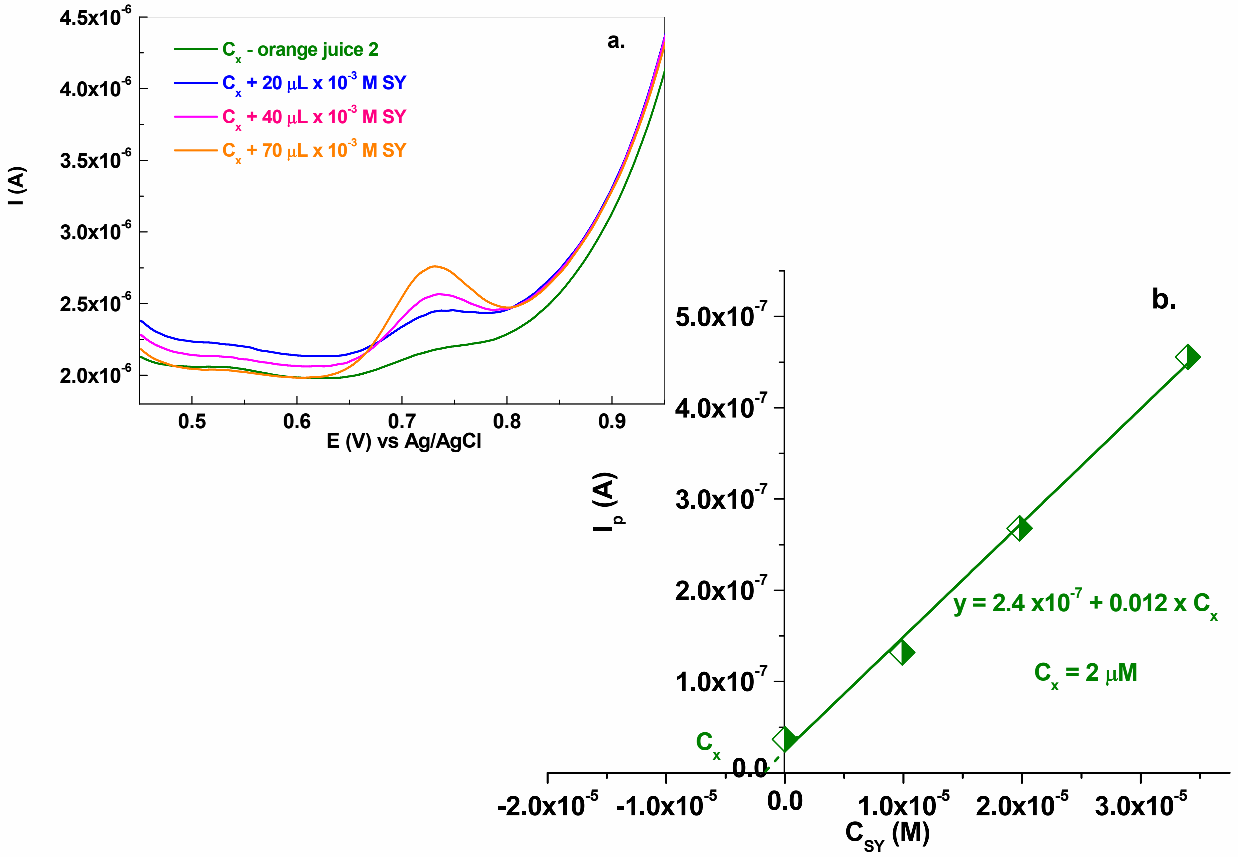

Figure S9. (**a**) Square wave voltammograms recorded with GR-exf/GC modified electrode in solutions containing orange juice 2; (**b**) the standard addition plot which allowed the determination of C_x in the second orange juice solution.
